# A very low incidence of *BRAF* mutations in Middle Eastern colorectal carcinoma

**DOI:** 10.1186/1476-4598-13-168

**Published:** 2014-07-08

**Authors:** Abdul K Siraj, Rong Bu, Sarita Prabhakaran, Prashant Bavi, Shaham Beg, Mohsen Al Hazmi, Maha Al-Rasheed, Khadija Alobaisi, Fouad Al-Dayel, Hadeel AlManea, Nasser Al-Sanea, Shahab Uddin, Khawla S Al-Kuraya

**Affiliations:** 1 Human Cancer Genomic Research, Research Center, King Faisal Specialist Hospital and Research Center, MBC#98-16, P.O. Box 3354 Riyadh 11211, Saudi Arabia; 2 Colorectal Unit, Department of Surgery, King Faisal Specialist Hospital and Research Center, Riyadh, Saudi Arabia; 3 Department of Pathology, King Faisal Specialist Hospital and Research Center, Riyadh, Saudi Arabia; 4 Department of Pathology, College of Medicine, Alfaisal University, Riyadh, Saudi Arabia

**Keywords:** Colorectal cancer, *BRAF* mutation, *KRAS* mutation, Microsatellite instability (MSI), Lynch Syndrome (LS), Hereditary non polyposis colorectal cancer (HNPCC) syndrome

## Abstract

**Background:**

Recent studies emphasize the role of *BRAF* as a genetic marker for prediction, prognosis and risk stratification in colorectal cancer. Earlier studies have reported the incidence of *BRAF* mutations in the range of 5-20% in colorectal carcinomas (CRC) and are predominantly seen in the serrated adenoma-carcinoma pathway characterized by microsatellite instability (MSI-H) and hypermethylation of the MLH1 gene in the setting of the CpG island methylator phenotype (CIMP). Due to the lack of data on the true incidence of *BRAF* mutations in Saudi Arabia, we sought to analyze the incidence of *BRAF* mutations in this ethnic group.

**Methods:**

770 CRC cases were analyzed for *BRAF* and *KRAS* mutations by direct DNA sequencing.

**Results:**

*BRAF* gene mutations were seen in 2.5% (19/757) CRC analyzed and *BRAF* V600E somatic mutation constituted 90% (17/19) of all *BRAF* mutations. *BRAF* mutations were significantly associated with right sided tumors (p = 0.0019), MSI-H status (p = 0.0144), CIMP (p = 0.0017) and a high proliferative index of Ki67 expression (p = 0.0162). Incidence of *KRAS* mutations was 28.6% (216/755) and a mutual exclusivity was noted with *BRAF* mutations (p = 0.0518; a trend was seen).

**Conclusion:**

Our results highlight the low incidence of *BRAF* mutations and CIMP in CRC from Saudi Arabia. This could be attributed to ethnic differences and warrant further investigation to elucidate the effect of other environmental and genetic factors. These findings indirectly suggest the possibility of a higher incidence of familial hereditary colorectal cancers especially Hereditary non polyposis colorectal cancer (HNPCC) syndrome /Lynch Syndrome (LS) in Saudi Arabia.

## Introduction

A complex network of genes are required for maintaining the cellular homeostasis in colorectal tissue. In the original multistep progression model of colorectal cancer (CRC) first proposed by Vogelstein et al, normal colonic epithelium gets transformed into benign (adenoma) neoplastic epithelium followed by full blown invasive cancer and eventually metastasis [1]. Over the years, significant advances in molecular genetics and caner biology have led to the understanding that there exist 3 distinct molecular phenotypes of CRC: (1) chromosomal instability(CIN) [2]; (2) microsatellite instability(MSI) [3]; and (3) the recently discovered sessile and traditional serrated adenomas, a subset of hyperplasic polyps that progress to serrated carcinomas [4].

Some of the key signaling pathways implicated in colorectal carcinogenesis are Epidermal Growth Factor Receptor Signaling (EGFR) pathway, WNT signaling [1] and transforming growth factor-β (TGF β) signaling [5]. Translational research has resulted in the bench-to-bedside application of biomarkers like *KRAS*, *BRAF* and PI3K for personalized medicine [6]. One of the key determinants of response to panitumumab and cetuximab therapy in colorectal cancer is the presence of mutations in the *KRAS* gene [7,8]. Testing for certain “activating” mutations in the *KRAS* gene is currently one of the most widely employed methods to predict responsiveness to EGFR inhibitors. Patients with presence of *KRAS* mutations in their tumors do not respond to EGFR inhibitors. On the other hand, a significant proportion of CRC patients with the wild-type (normal) *KRAS* gene fail to respond to EGFR inhibitors and mutations in other genes such as *PIK3CA/BRAF/NRAS/PTEN/TP53* have been implicated for resistance in this subgroup of patients [7-9]. *BRAF* mutations are known to be mutually exclusive with *KRAS* mutations and Vaughn et al have reported that almost 8% of the CRC subgroup with wild type *KRAS* gene had *BRAF* mutations; these patients would receive EGFR inhibitors but would be unresponsive to therapy [8].

Constitutive activation of the mitogen-activated protein kinase (MAPK) pathway regulates cellular proliferation, differentiation, and death. Oncogenic mutations in the v-Raf murine sarcoma viral oncogene homolog B1 (*BRAF)* in CRC were first described by Davies et. al and the commonest mutation was a single phosphomimetic substitution in the kinase activation domain (V600E) that led to activation of the MAPK pathway [10] Although *BRAF* mutations have been identified in a variety of cancers, their highest incidence to the tune of almost 60% is seen in melanomas [10]. The CRC subset that originates from advanced serrated polyps have a distinct molecular phenotype characterized by widespread hypermethylation of CpG islands in the promoter regions of genes, referred to as the CpG island methylator phenotype (CIMP) [11], microsatellite instability (MSI) and oncogenic mutations in *BRAF* gene [4,12,13]. Testing for *BRAF* mutation is beneficial in judicious selection of patients for targeted therapy [8,9] and also a cost effective approach in the HNPCC (Hereditary Non-polyposis Colorectal Cancer) workup; presence of *BRAF* mutation with absence of MLH1 protein is indicative of sporadic CRC [14,15].

In this study we comprehensively investigated the incidence of *BRAF* mutations in Saudi CRC and its clinico-pathological correlation, its association with molecular markers and overall survival.

## Materials and methods

### Patients’ selection and TMA construction

A total of 770 patients with CRC diagnosed between 1990 and 2011 were randomly selected from King Faisal Specialist Hospital and Research Centre (KFSHRC), and Security Forces Hospital (SFH), Riyadh. A colorectal tissue microarray was constructed comprising of 770 CRC samples as described previously [16]. Clinical and histopathological data were available for all these patients. Patients with colon cancer underwent surgical colonic resection and those with rectal cancer underwent anterior resection or abdominoperineal resection. All node-positive colon cancers received 5-fluorouracil-based adjuvant chemotherapy. A vast majority of the rectal cancers received radiotherapy alone or chemoradiotherapy prior to surgery, followed by adjuvant chemotherapy after surgery. Colorectal Unit, Department of Surgery (KFSHRC and SFH), provided long-term follow-up data about the date and cause of death for this cohort of patients. Follow-up was calculated from the date of resection of the primary tumor, and all surviving cases were censored for survival analysis on 31st December 2011. Two pathologists (P.B., S.P.) reviewed all tumors for grade and histological subtype. The institutional review board of the King Faisal Specialist Hospital and Research Centre approved the study.

### DNA isolation

DNA was extracted from paraffin-embedded CRC tissues using Gentra DNA isolation kit (Gentra, Minneapolis, MN, USA) following the manufacturer’s recommendations as described previously [17].

### PCR and DNA sequencing for *KRAS* and *BRAF* gene

*KRAS* and *BRAF* mutation analysis was performed on 755 and 757 CRC samples respectively. Primer 3 software was used to design the primers for Exon 15 of *BRAF*; Exon 1, 2 of *KRAS* (Table 1). The PCR sequencing protocol was same as described earlier [17]. The samples were finally analyzed on an ABI PRISM 3100xl genetic analyzer (Applied Biosystems, Foster City, CA).

**Table 1:** **Primer sequence of *BRAF *and *KRAS *gene**

**Exon**	**Forward primer**	**Reverse primer**
***BRAF***		
Exon 15	TGCTTGCTCTGATAGGAAAATG	AGCATCTCAGGGCCAAAAAT
***KRAS***		
Exon 1	TTAACCTTATGTGTGACATGTTCTAA	AGAATGGTCCTGCACCAGTAA
Exon 2	CCAGACTGTGTTTCTCCCTTC	TTTAAACCCACCTATAATGGTGAA

### Microsatellite markers and analyses

Allelic imbalances were measured by performing microsatellite analysis on all matched normal and tumor tissue by PCR amplification. A reference panel of five pairs of microsatellite primers, comprising two mononucleotide microsatellites (BAT25, BAT26) and three dinucleotide microsatellites (DS123, D5S346 and D17S250) were used to determine tumor MSI status [3]. Multiplex PCR was performed in a total volume of 25 μl using 50 ng of genomic DNA, 2.5 μl 10 × Taq buffer, 1.5 μl MgCl2 (25 mM), 10 pmol of fluorescent-labeled primers, 0.05 μl dNTP (10 mM) and 0.2 μl Taq polymerase (1 Uμl − 1; all reagents were from Qiagen Inc., Valencia, CA, USA). PCR was performed using an MJ Research PTC-200 thermocycler. The samples in which the novel alleles were found at one, and two or more of those five loci were assigned MSI-L and MSI-H respectively, and whereas samples without novel alleles at any one of those loci were assigned MSS.

### Statistical analysis

The JMP 10.0 (SAS Institute Inc., Cary, NC) software package was used for data analyses. Survival curves were generated using Kaplan-Meier method, with significance evaluated using the Mantel-Cox log-rank test. Risk ratio was calculated using the Cox proportional hazard model in both univariate and multivariate analyses. Values of p < 0.05 were considered statistically significant.

## Results

### Clinico-pathologic data

The characteristics of the 770 CRC patients are described earlier [18]. The median age at the time of surgery was 57 years (inter quartile range [IQR], 47.7 -68.0 years). The 5 year overall survival of our study population was 70.6%.

### *BRAF* mutation and their clinico-pathological correlation

**Figure 1:**
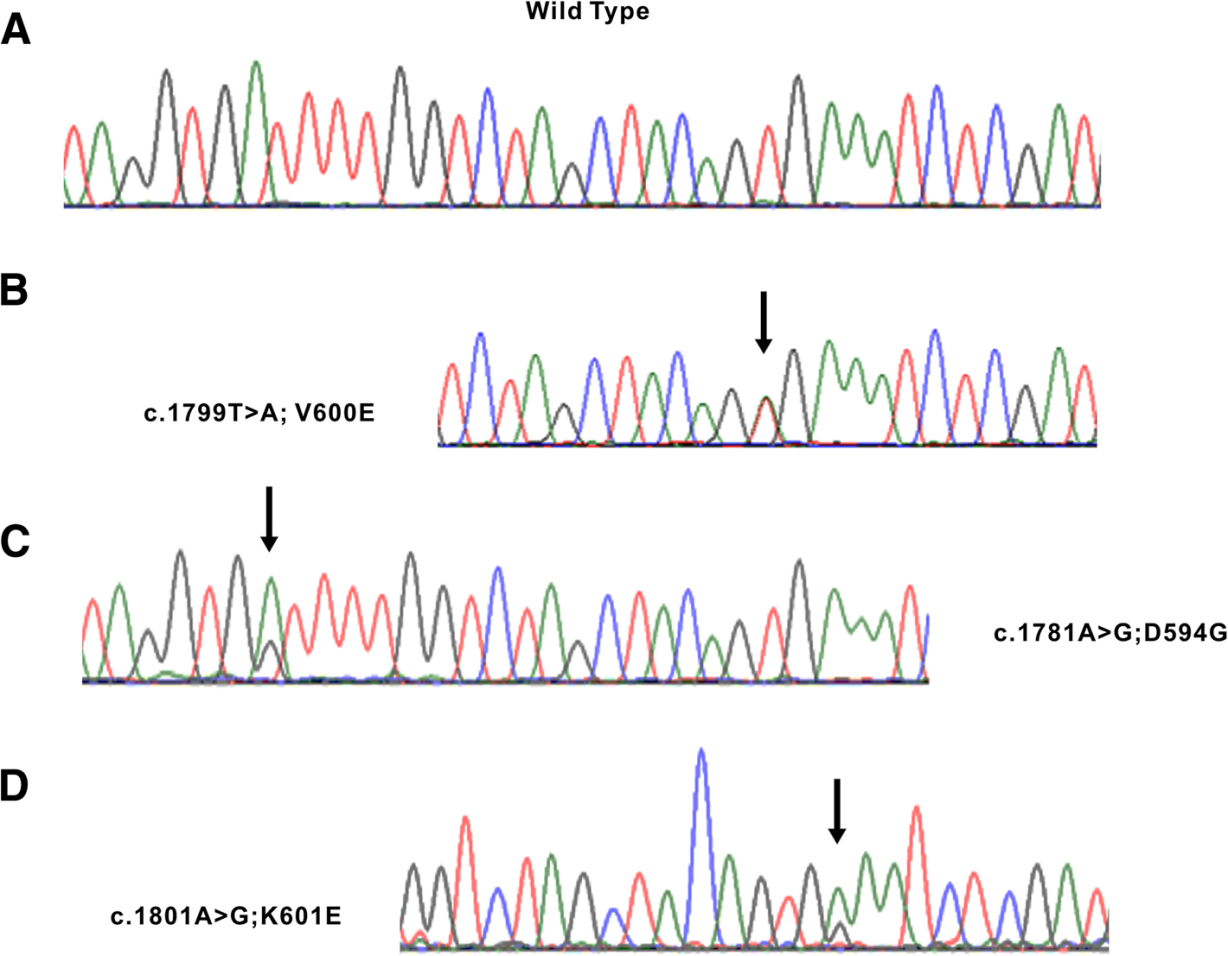
**Example of *BRAF *gene mutations in CRC.** Sequencing traces of cases harboring wild type **(A)**, and V600E **(B)**, D594G **(C)** and K601E **(D)** mutations, respectively.

Seven hundred fifty seven colorectal cancer cases were analyzed for *BRAF* mutations and the remaining 13 cases were not interpretable due to insufficient amount of DNA and other technical reasons. Of the 757 cases analyzed for *BRAF* status, *BRAF* mutations were observed in nineteen cases and the incidence of *BRAF* in Saudi colorectal cancer was 2.5%( 19/757) (Table 2). Surprisingly this is among the lowest incidence of *BRAF* mutations reported in literature (Tables 3 and 4). Of these 19 CRC with *BRAF* mutations, 17 were seen in the V600E type and the other 2 were seen in V594G and V601E (Table 3 and Figure 1). To reconfirm these results and rule out the fact that we were missing any *BRAF* mutations due to tumor heterogeneity issues, cancer tissue was re-punched from 2-3 different tumor areas and *BRAF* analysis was repeated on 400 of these 757 samples. Repeat Sanger sequencing did not show any discordance with earlier results and failed to reveal any new cases with *BRAF* mutation. As shown in Table 2, CRC with *BRAF* mutations were significantly associated with right sided tumors (p = 0.0019), microsatellite instable MSI-H status (p = 0.0144) and CIMP high phenotype (p = 00017). Of the 19 cases with *BRAF* mutation, MSI-H, MSI-L and MSS were 6 cases (31.6%), 5 cases (26.3%) and 8(42.1%) cases respectively. The degree of Ki-67 staining as a measure of proliferative index was significantly higher in the *BRAF* mutation positive CRC group (88.89 ± 12.78) as compared to the *BRAF* mutation negative group (80.60 ± 25.30, p = 0.0162; Students T test; Additional file 1: Figure S1). A statistical trend was noted with presence of *BRAF* mutation and older age (age > 50; p = 0.0938) and a mutual exclusivity was observed with *KRAS* mutations (p = 0.0518; a statistical trend was noted). *BRAF* mutations were not associated with gender, histology subtype, tumor differentiation and Stage.

**Table 2:** **Correlation of *BRAF *Mutation with clinico-pathological parameters in colorectal carcinoma**

	**Total**		**Positive**		**Negative**		**P value**
	**N**	**%**	**N**	**%**	**N**	**%**	
**Total number of cases**	757		19	2.5	738	97.5	
**Age**							
< 50 years	246	32.5	3	1.2	243	98.8	0.0938
> 50 years	511	67.5	16	3.1	495	97.9	
**Sex**							
Male	394	52.0	11	2.8	383	97.2	0.6044
Female	363	48.0	8	2.2	355	97.8	
Tumour site*							
Left colon	600	83.0	10	1.7	590	98.3	0.0019
Right colon	123	17.0	9	7.3	114	92.7	
**Histological type**							
Adenocarcinoma	673	88.9	18	2.7	655	97.3	0.3673
Mucinous Carcinoma	84	11.1	1	1.2	83	98.8	
Tumour stage*							
I	88	12.2	4	4.6	84	95.4	0.6853
II	255	35.3	7	2.7	248	97.3	
III	289	40.0	6	2.1	283	97.9	
IV	90	12.5	2	2.2	88	97.8	
**Differentiation**							
Well	74	9.8	2	2.7	72	97.3	0.8887
Moderate	590	77.9	14	2.4	576	97.6	
Poor	93	12.3	3	3.2	90	96.8	
MSI-Molecular*							
MSI-H	81	11.1	6	7.4	75	92.6	0.0144
MSI-S/L	651	88.9	13	2.0	638	98.0	
***KRAS *** Mutation*							
Positive	216	28.7	2	0.9	214	99.1	0.0518
Negative	537	71.3	17	3.2	520	96.8	
CIMP*							
High	24	5.1	4	16.7	20	83.3	0.0017
Low & middle	444	94.9	8	1.8	436	98.2	

*Data were not available (NA) for some cases for tumor site (NA = 34), Stage (NA = 35), MSI-Molecular (NA = 25), *KRAS* Mutation (NA = 4), and CIMP (NA = 289).

**Table 3:** **Clinical information, pathologic diagnosis, *BRAF *and *KRAS *testing results**

**Case #**	**AGE**	**Gender**	**Histology Type**	**Site**	**Grade**	**Stage**	***BRAF***	***KRAS***	**CIMP**	**MSI-PCR**	**Pre-existing**
											**Adenoma**
*BRAF*-1	60	Male	AC	Caecum	2	I	V601E	Codon 13	Low CIMP	MSI-L	Tubular adenoma
*BRAF*-2	47	Male	AC	Ascending colon	2	I	V600E	Codon 13	NA	MSI-S	Hyperplastic polyp
											SERRATED ADENOMA
*BRAF*-3	71	Female	AC	Rt colon	1	III	V600E	WT	NA	MSI-H	Unremarkablemucosa
*BRAF*-4	57	Female	AC	Rt colon	3	IV	V600E	WT	Low CIMP	MSI-S	Unremarkablemucosa
*BRAF*-5	67	Male	AC	Recto sigmoid	2	II	V600E	WT	Low CIMP	MSI-S	Unremarkablemucosa
*BRAF*-6	74	Female	AC	Rt colon	3	II	V600E	WT	Low CIMP	MSI-H	Unremarkablemucosa
*BRAF*-7	66	Male	AC	Ascending colon	2	III	V594G	WT	Low CIMP	MSI-L	Adenoma
*BRAF*-8	68	Male	AC	Rt colon	2	IV	V600E	WT	Low CIMP	MSI-S	Unremarkablemucosa.
*BRAF*-9	61	Female	AC	Rt colon	1	I	V600E	WT	High CIMP	MSI-H	Small hyperplasticpolyps
*BRAF*-10	46	Male	AC	Sigmoid	2	I	V600E	WT	NA	MSI-L	Hyperplastic polyp
*BRAF*-11	34	Male	AC	Recto sigmoid	2	III	V600E	WT	Low CIMP	MSI-S	Unremarkablemucosa
*BRAF*-12	71	Male	AC	Sigmoid	2	III	V600E	WT	NA	MSI-S	Hyperplastic polyp
*BRAF*-13	59	Male	AC	Rectal	2	III	V600E	WT	High CIMP	MSI-S	Tubular adenoma
*BRAF*-14	66	Female	AC	Rt colon	2	II	V600E	WT	NA	MSI-H	Unremarkable mucosa
*BRAF*-15	66	Male	AC	Sigmoid colon	2	II	V600E	WT	Low CIMP	MSI-S	No colonic mucosa seen
*BRAF*-16	73	Female	AC	Rt colon	3	II	V600E	WT	High CIMP	MSI-H	Unremarkable mucosa
*BRAF*-17	72	Male	AC	Rt colon	2	II	V600E	WT	High CIMP	MSI-H	Unremarkable mucosa
*BRAF*-18	74	Female	AC	Sigmoid colon	2	II	V600E	WT	NA	MSI-L	Unremarkable mucosa
*BRAF*-19	55	Female	MC	Recto sigmoid	2	III	V600E	WT	NA	MSI-L	Unremarkable mucosa

**Table 4:** **Summary of previous studies on *BRAF *mutation in colorectal carcinoma**

**Study**	**Country**	**% with *BRAF***	***BRAF *** **mutation detected**	**Methods of mutation detection**
**Li-Ling H, 2012**	Taiwan	1.1	2/182	High-resolution melting point (HRM) polymerase chain reaction (PCR) for *BRAF* V600E mutations
**Li H , 2011**	China	7	14/200	Sequenced by Pyrosequencer PyroMark ID system-Exon 15, V600E
**Liou J, 2011**	China	3.8	12/314	Sequencing of exons 11 and 15
**Yokota T, 2011**	Japan	4.7	15/319	Cycleave PCR technique for V600E mutation
**Nakanishi R, 2012**	Japan	6.7	17/254	*BRAF* pyrokit pyrosequencing
**Ajay Goel, 2009**	Israel	18.7	24/128	Direct sequencing using the BigDye version 1.1 cycle-sequencing kit
**Rozek L, 2010**	Israel	5	65/1300	Direct sequencing of exon 15 of *BRAF*
**Price T, 2011**	Australia	10.6	33/315	High-resolution melting point (HRM) polymerase chain reaction (PCR) for *BRAF* V600E mutations
**Tran B, 2011**	Australia	11	57/524	Mutation-specific real-time polymerase chain reaction assay.
**Di Nicolantonio, 2008**	Italy	10	11/113	Automated sequencing by ABI PRISM 3730 exon15
**Richman S, 2009**	UK	7.9	422/711	Pyrosequenced on a PyroMark ID system (Biotage AB) Codon 600
**Roth A, 2009**	Switzerland	7.9	103/1307	Allelic discrimination assay on a 7500 real-time polymerase chain reaction for V600E
**Zlobec I, 2010**	Switzerland	12	45/374	*BRAF* (exon 15 codon 600) direct sequencing of single-stranded PCR products using the BigDyeVR Terminator v1.1 cycle sequencing kit
**Farina-Sarasqueta A, 2010**	Netherlands	19.8	59/297	V600E mutation on the *BRAF* gene by real-time PCRLight Cycler v2.0 (Roche)
**Saridaki Z, 2010**	Greece	8.3	12/144	Real-time PCR using the allelic discrimination method with ABI PRISM 7900 T Sequence Detection System
**Modest D, 2012**	Germany	11.6	17/146	Exons 11 and 15 of the *BRAF*pyro-sequencing
**Samowitz W, 2005**	USA	9.5	87/911	Sequencing in both directions
**Shaukat A,2010**	USA	21.8	36/165	*BRAF* V600E mutation -Mutector II *BRAF*/Ras mutation detection panel assay kit
**Kalady M,2012**	USA	12	56/475	Direct sequencing
**Ogino S,2012**	USA	15	11/75	c.1799 T > A (p.V600E) mutation

All the pathology reports of colonic biopsies and resection specimens and slides were reviewed to ascertain the presence of serrated adenomas in the 19 cases with *BRAF* mutations. We confirmed the presence of a serrated adenoma in only 1 case; hyperplastic polyps in 3 cases; tubular adenomas in 2 and the remaining 13 cases showed unremarkable colorectal mucosa.

### *KRAS* mutation and their clinico-pathological correlation

In our series of 770 CRC samples 755 were analyzable for *KRAS* mutations and the incidence of *KRAS* mutations was 28.6% (216/755; Additional file 2: Table S1). The remaining 15 samples of the 770 CRC could not be analyzed due to insufficient DNA or technical reasons. Most of *KRAS*-mutated cases showed a mutation at codon 12.

(152 cases; 70.3%) and codon 13 (64 cases; 29.7%). *KRAS* mutations were associated with right-sided CRC (p = 0.0064).

### Co-existence of *KRAS* and *BRAF* mutation

The clinico-pathological and molecular characteristics of the two CRC cases with co-existence of *KRAS* and *BRAF* mutations - “*BRAF*-1” and “*BRAF*-2” are summarized in Table 3. Both the patients were males with age > 40 years(47 years and 60 years); MSI-L/MSS type; histology subtype of adenocarcinomas with moderate differentiation; and Stage I cancer. Both these cases had mutation in codon 13 for *KRAS* gene and the *BRAF* gene showed mutation at V600E and V601E for *BRAF*-1 and *BRAF*-2 specimen respectively.

### Microsatellite instability analysis

MSI analysis data was available in 741 of the 770 samples and the incidence of microsatellite instable (MSI-H), microsatellite low (MSI-L) and microsatellite stable (MSS) was 11.2%( 83/741), 18.6% (138/741) and 70.2% ( 520/741) respectively. The remaining 29 samples could not be analyzed due to insufficient DNA, lack of paired normal DNA or technical reasons.

### *BRAF* mutation, *KRAS* mutation, and overall survival

**Figure 2:**
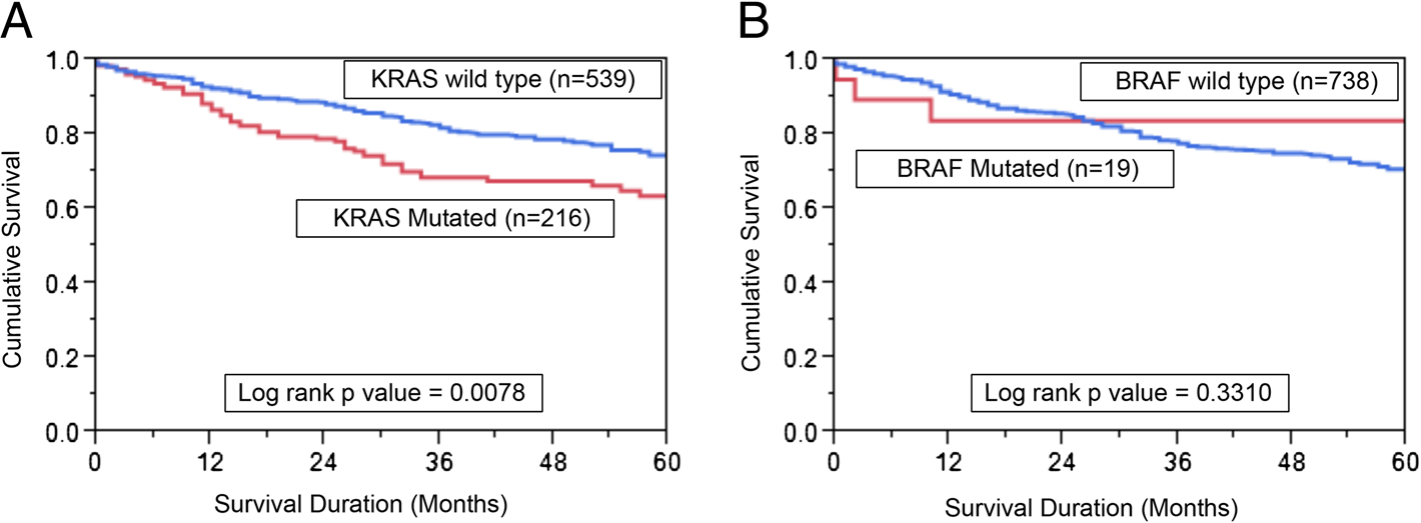
**Impact of *KRAS *and *BRAF *mutations in CRC and the Kaplan–Meier.** Survival analysis. **(A)** Colorectal cancer patients with *KRAS* mutations had reduced overall survival of 63.5% at 5 years compared with 73.5% without *KRAS* mutations (p = 0.0078). **(B)** In CRC patients, there was no significance in survival between *BRAF* mutated and non mutated cases. (p = 0.3310).

The prognostic significance of *KRAS* and *BRAF* mutation was analyzed with the use of the Kaplan–Meier method. Patients with *KRAS* mutation had poorer survival as compared to CRC with wild type *KRAS* gene (p = 0.0078) (Figure 2A). In the multivariate analysis using the Cox proportional hazard model (Additional file 3: Table S2) for multiple factors such as age, gender, AJCC stage, microsatellite instability and tumor differentiation, the relative risk was 1.75 for CRC with *KRAS* mutation(95% CI 1.26-2.42; p = 0.0011) and 6.70 for advanced AJCC stage(95% CI 4.78-9.31; p ≤ 0.0001). Thus, *KRAS* mutation was an independent prognostic marker for poor survival in CRC across all stages. We also assessed the overall survival of *KRAS* mutation at codon12 and codon13. Amongst the *KRAS*-mutated cases, mutation in codon 12 was associated with the worst survival (62.8%; *p* = 0*.*0230) compared with codon 13 mutations (64.7%) or absence of *KRAS* mutations (73.5%). *BRAF* mutation was not associated with any prognostic significance (p = 0.3310; Figure 2B).

## Discussion

Accumulating evidence implicates *BRAF* mutations to have diagnostic, therapeutic and prognostic significance in CRC [10,19-22]. In our study, the *BRAF*-V600E mutation was identified in only 2.5% of all CRC, which is significantly lower than earlier reports of *BRAF* mutation in CRC worldwide (5-15%; [22-26]). The overall low frequency of the *BRAF*-V600E mutation and serrated adenomas in this unique ethnic group of Saudi colorectal cancer cases suggests a very small role of the *BRAF* gene in the development of CRC and raises some interesting future questions on the burden of familial colorectal cancers like Hereditary non polyposis colorectal cancer cancer(HNPCC) syndrome in Saudi Arabia.

The reported frequency of *BRAF* mutations in different populations varies widely from a low incidence of 1% in Taiwan to 19.8% and 21.8% in Netherlands and USA respectively [22-25,27-40]. A lower frequency has been observed in most of the Asian population as is evident from the lowest *BRAF* mutation incidence of 1% in Taiwan where *BRAF* mutation was seen in 2/182 CRC. A similar low incidence was seen in China (3.8% and 7%) and Japan (4.7% and 6.7%). *BRAF* mutation was also observed at a lower rate of 5% in Israel [39]. Interestingly the frequency of the *BRAF* V600E mutation varies widely between population groups, both within single studies (5.8% in the Ashkenazi versus 3.2% in the non-Ashkenazi) and also between reports from different groups but within the same region/country [36,41]. The lower incidence of *BRAF* mutations in Saudi CRC patients could be due to a different ethnic populations with varied underlying genetic predisposition to *BRAF*-mutated tumors, role of environmental influences like diet, smoking and other unknown factors. Different methods used to detect gene mutation such as 454 next generation sequencing, Sanger sequencing, pyrosequencing and melting curve analysis also influence the detection rate of a gene mutation [42]. An earlier study has highlighted the differing rates of *BRAF* mutation in distinct ancestral populations with a lower frequency observed in Asian population as compared to White and Black patients in their study [43].

Although *BRAF* mutations occur early in colorectal carcinogenesis and have been observed in colorectal adenomas, tumor progression needs additional acquired DNA microsatellite instability caused by hypermethylation of *MLH1* in the setting of the CpG island methylator phenotype (CIMP) [12,44]. These precursor lesions that harbor *BRAF* mutation, microsatellite instability and CIMP phenotype have a distinct morphology and are termed serrated adenomas. A retrospective review of all the available biopsies of hyperplastic polyps and adenomas biopsies confirmed the presence of only one case that had a morphology consistent with serrated adenomas. Additional efforts to identify serrated adenomas from the FFPE archives of Department of Pathology confirmed the fact that serrated adenomas were exceedingly rare. We have analyzed CIMP in 500 CRC patients and have observed a very low frequency of CIMP of 4.8% (data not shown). In concordance with an earlier study we hypothesize that lack of a propensity for hypermethylation and other factors in Saudi CRC prevents the driver *BRAF* mutation in premalignant lesions to progress to full blown cancers [13]. Although smoking is one of the factors implicated in CIMP phenotype and the incidence of smoking in Saudi Arabia is quite high [45,46], the prevalence of *BRAF* mutations, CIMP and serrated adenomas in our study population was on the lower side. Moreover, the significantly lower incidence of *BRAF* mutations in our study suggests the existence of a possibly higher number of familial syndromes like HNPCC that are characterized by a virtual lack of *BRAF* abnormalities in colorectal cancers. A study is being conducted to determine the true incidence of HNPCC in Saudi Arabia. Presence of *BRAF* V600E mutation in CRC tumors that lacks the expression of MLH1 by IHC does not warrant further genetic testing and excludes the possibility of the presence of a germline mutation in mismatch repair (MMR) genes. This exquisite sensitivity of *BRAF* V600E somatic mutation for sporadic MSI has led to the development of an algorithm for screening of LS as a multistep diagnostic approach in several guidelines and recommendations [15,47]. Since a significant proportion of sporadic CRC get excluded, the use of *BRAF* V600E as a screening tool to identify sporadic MSI CRC tumors is highly cost effective [15,47,48]. Newer developments in the detection of *BRAF* V600E mutation in CRC by IHC have shown to have a comparable sensitivity and specificity as PCR testing [49]. After stringent validation of *BRAF* IHC in each lab, *BRAF* testing by IHC will prove to be a simple, economical, labor and time saving test that can be performed even in small biopsies that yield a small amount of DNA. Finally Weisenberger et al investigated CIMP in CRC and demonstrated that MSI-H CRC are either HNPCC or MSI-H and CIMP + with or without MLH1 methylation [50]. The authors concluded that CIMP + encompass almost all sporadic MSI-H CRC while MLH1 methylation constitute a part of, but not all CIMP + CRC tumors. Since *BRAF* mutations distinctly correlate with CIMP + CRC, *BRAF* testing could outperform MLH1 methylation in identifying sporadic MSI-H CRC tumors in the diagnostic approach of LS [50].

Emerging studies highlight the critical significance of the impact of *BRAF* mutations on colorectal research. Although there has been remarkable success in using *BRAF* inhibitors in melanomas with a response rate of over 80%, a lower response rate of about 10% is seen in CRCs [20]. A key mechanism causing resistance to *BRAF* inhibitors in CRC is upregulation of the EGFR pathway and a combination therapy with EGFR inhibitors and *BRAF* inhibitors might prove effective [20]. Dual synergism could guide strategies that aim to improve outcomes especially in patients who are refractory to initial lines of therapy.

In conclusion, we observed a very low incidence of CRC with *BRAF* mutations that showed a significant association with right sided tumors, CIMP high phenotype and microsatellite instability. Future work would be directed to establish an effective screening program to study the prevalence of HNPCC cases in Saudi Arabia and also try to understand the complex interaction between genetics and environmental factors that contribute to this low incidence of *BRAF* mutations in this region.

### Consent

Waiver of consent was obtained for the study from Research Ethic committee under Project RAC 2080 030 on archival colorectal samples.

## Competing interest

This manuscript has not been published earlier and the authors have declared no competing interest.

## Authors’ contribution

PB, RB, SP & SB acquisition of data; analysis and interpretation of data; drafting of the manuscript; critical revision of the manuscript for important intellectual content; statistical analysis; technical support. MAR, MAH and KAO acquisition of data; analysis and interpretation of data; drafting of the manuscript; technical support. AKS, NAS, FAD, HAM and SU acquisition of data; analysis and interpretation of data, critical revision of the manuscript for important intellectual content technical, or material support; study supervision. KSA study concept and design; analysis and interpretation of data; drafting of the manuscript; critical revision of the manuscript for important intellectual content; study supervision. All authors read and approved the final manuscript.

## Supplementary Material

Additional file 1: Figure S1 *BRAF* mutation and proliferation index as measured by Ki-67 IHC expression. Box Plot charts indicates the mean and SD of Ki-67 expression in two groups: The expression of Ki-67 in *BRAF* Positive (88.89 ± 12.78) and *BRAF* Negative (80.60 ± 25.30, p value 0.0162). Click here for file

Additional file 2: Table S1 Correlation of KRAS Mutation with clinico-pathological parameters in colorectal carcinoma. Click here for file

Additional file 3: Table S2 Univariate and Multivariate analysis of Kras Mutation using Cox Proportional Hazard Model. Click here for file
