# Supplementary figures and images for: A very low incidence of BRAF mutations in Middle Eastern colorectal carcinoma
Source: Mol Cancer. 2014 Jul 8;13:168. doi: 10.1186/1476-4598-13-168 (PMC4109832; doi:10.1186/1476-4598-13-168)

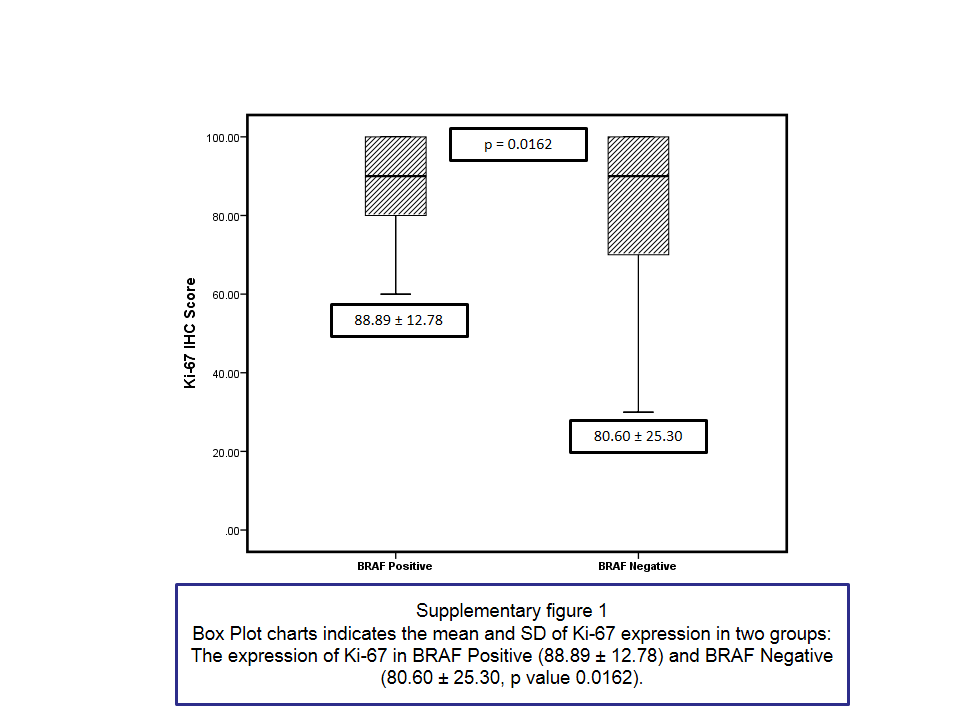

Supplement: Additional file 1: Figure S1 — BRAF mutation and proliferation index as measured by Ki-67 IHC expression. Box Plot charts indicates the mean and SD of Ki-67 expression in two groups: The expression of Ki-67 in BRAF Positive (88.89 ± 12.78) and BRAF Negative (80.60 ± 25.30, p value 0.0162). [file 1476-4598-13-168-S1.tiff]
