# Supplementary material for: A very low incidence of BRAF mutations in Middle Eastern colorectal carcinoma
Source: Mol Cancer. 2014 Jul 8;13:168. doi: 10.1186/1476-4598-13-168 (PMC4109832; doi:10.1186/1476-4598-13-168)
Supplement: Additional file 2: Table S1 — Correlation of KRAS Mutation with clinico-pathological parameters in colorectal carcinoma. [file 1476-4598-13-168-S2.docx]

Additional file 2: Table S1. Correlation of KRAS Mutation with clinico-pathological parameters in colorectal carcinoma

|  | **Total** | | **Positive** | | **Negative** | | **P value** |
| --- | --- | --- | --- | --- | --- | --- | --- |
|  | **N** | **%** | **N** | **%** | **N** | **%** |  |
| **Total Number of Cases** | 755 |  | 216 | 28.6 | 239 | 71.4 |  |
| **Age** |  |  |  |  |  |  |  |
| < 50 years | 246 | 32.6 | 71 | 28.9 | 175 | 71.1 | 0.9150 |
| > 50 years | 509 | 67.4 | 145 | 28.5 | 364 | 71.5 |  |
| **Sex** |  |  |  |  |  |  |  |
| Male | 395 | 52.3 | 110 | 27.8 | 285 | 72.1 | 0.6279 |
| Female | 360 | 47.7 | 106 | 29.4 | 254 | 70.6 |  |
| **Tumour Site*** |  |  |  |  |  |  |  |
| Left colon | 599 | 83.1 | 156 | 26.0 | 443 | 74.0 | 0.0064 |
| Right colon | 122 | 16.9 | 47 | 38.5 | 75 | 61.5 |  |
| **Histological Type** |  |  |  |  |  |  |  |
| Adenocarcinoma | 671 | 88.9 | 186 | 27.7 | 485 | 72.3 | 0.1337 |
| Mucinous Carcinoma | 84 | 11.1 | 30 | 35.7 | 54 | 64.3 |  |
| **Tumour Stage*** |  |  |  |  |  |  |  |
| I | 88 | 12.2 | 28 | 31.8 | 60 | 68.2 | 0.6815 |
| II | 255 | 35.4 | 67 | 26.3 | 188 | 73.7 |  |
| III | 288 | 39.9 | 86 | 29.9 | 202 | 70.1 |  |
| IV | 90 | 12.5 | 24 | 26.7 | 66 | 73.3. |  |
| **Differentiation** |  |  |  |  |  |  |  |
| Well | 74 | 9.8 | 29 | 39.2 | 45 | 60.8 | 0.0339 |
| Moderate | 588 | 77.9 | 155 | 26.4 | 433 | 73.6 |  |
| Poor | 93 | 12.3 | 32 | 34.4 | 61 | 65.6 |  |
| **MSI-Molecular*** |  |  |  |  |  |  |  |
| MSI-H | 82 | 11.2 | 23 | 28.0 | 59 | 72.0 | 0.9443 |
| MSI-S/L | 651 | 88.8 | 185 | 28.4 | 466 | 71.6 |  |
| **Braf Mutation*** |  |  |  |  |  |  |  |
| Positive | 19 | 2.5 | 2 | 10.5 | 17 | 89.5 | 0.0518 |
| Negative | 734 | 97.5 | 214 | 29.2 | 520 | 70.8 |  |
| **CIMP*** |  |  |  |  |  |  |  |
| High | 15 | 3.2 | 4 | 26.7 | 11 | 73.3 | 0.7806 |
| Low & Middle | 447 | 96.8 | 134 | 30.0 | 313 | 70.0 |  |
| **Survival** |  |  |  |  |  |  |  |
| OS 5 Years |  |  |  | 63.5 |  | 73.5 | 0.0078 |

*Data were not available (NA) for some cases for tumor site (NA=34), Stage (NA=34), MSI-Molecular (NA=22), KRAS Mutation (NA=2), CIMP (NA=293), and Ki-67 IHC (NA=46).
