# Supplementary material for: A very low incidence of BRAF mutations in Middle Eastern colorectal carcinoma
Source: Mol Cancer. 2014 Jul 8;13:168. doi: 10.1186/1476-4598-13-168 (PMC4109832; doi:10.1186/1476-4598-13-168)
Supplement: Additional file 3: Table S2 — Univariate and Multivariate analysis of Kras Mutation using Cox Proportional Hazard Model. [file 1476-4598-13-168-S3.docx]

Additional file 3: Table S2. Univariate and Multivariate analysis of Kras Mutation using Cox Proportional Hazard Model

| Clinical Parameters | **UNIVARIATE** | | **MULTIVARIATE** | |
| --- | --- | --- | --- | --- |
|  | Risk Ratio  (95% CI) | p value | Risk Ratio  (95% CI) | p value |
| **Age** Above>50 | 1.10 (0.81-1.51) | 0.5414 | 0.94 (0.68-1.32) | 0.7306 |
| **Sex** Male | 1.05 (0.78-1.41) | 0.7404 | 1.21 (0.89-1.66) | 0.2223 |
| **Stage** IV | 6.00 (4.39-8.16) | < 0.0001 | 6.70 (4.78-9.31) | < 0.0001 |
| **Grade** Poorly Diff. | 1.47 (0.98-2.14) | 0.0604 | 1.96 (1.28-2.92) | 0.0027 |
| **MSI** MSI-L/S | 1.33 (0.82-2.32) | 0.2552 | 1.15 (0.68-2.09) | 0.6194 |
| **Kras Mutation +** | 1.61 (1.16-2.21) | 0.0047 | 1.75 (1.26-2.42) | 0.0011 |
